# Supplementary material for: Cloning, phylogenetic research, and prokaryotic expression study of the metabolic detoxification gene EoGSTs1 in Empoasca onukii Matsuda
Source: PeerJ. 2019 Sep 6;7:e7641. doi: 10.7717/peerj.7641 (PMC6733243; doi:10.7717/peerj.7641)
Supplement: Supplemental Information 1 [file peerj-07-7641-s001.docx]

**Reaction system and conditions of 5′RACE PCR**

| **Table S1 First-round reaction system of 5′RACE PCR** | |
| --- | --- |
| Reagent | Volume (25 µL) |
| 5′RACE template | 0.5 µL |
| 5 GeneRacer outer primer (10 µM) | 1.0 µL |
| rGST-R1 (10 µM) | 1.0 µL |
| Platinum PCR Supermix High Fidelity | 22.5 µL |

| **Table S2 First-round reaction condition of 5′RACE PCR** | |
| --- | --- |
| Initial denaturation | 94°C 2 min |
| 5 cycles | 94°C 30sec, 72°C 30sec |
| 5 cycles | 94°C 30sec, 70°C 30sec |
| 25 cycles | 94°C 30sec, 66°C 30sec |

| **Table S3 Second-round reaction system of 5′RACE PCR** | |
| --- | --- |
| Reagent | Volume (25 µL) |
| First round product of 5′RACE PCR | 0.5 µL |
| 5 GeneRacer Inner primer (10 µM) | 1.0 µL |
| rGST-R2 (10 µM) | 1.0 µL |
| Platinum PCR Supermix High Fidelity | 22.5 µL |

| **Table S4 Second-round reaction condition of 5′RACE PCR** | |
| --- | --- |
| Initial denaturation | 94°C 2 min |
| 30 cycles | 94°C 30sec, 66°C 30sec |

**Reaction system and conditions of the middle fragment PCR**

| **Table S5 Reaction system** | |
| --- | --- |
| Reagent | Volume (50 µL) |
| Sterilized deionized water | 1.0 µL |
| mGST-F (10 µM) | 1.0 µL |
| mGST-R(10 µM) | 1.0 µL |
| cDNA template | 2.0 µL |
| Platinum® PCR SuperMix, High Fidelity | 45 µL |

| **Table S6 Reaction condition** | |
| --- | --- |
| Initial denaturation | 94°C 2 min |
| 38 cycles | 94°C 30 sec |
|  | 62°C 30 sec |
|  | 68°C 30 sec |

**Reaction system and conditions of 3′RACE PCR**

| **Table S7 First-round reaction system of 3′RACE PCR** | |
| --- | --- |
| Reagent | Volume (25 µL) |
| 3′RACE template | 0.5 µL |
| 3 GeneRacer outer primer (10 µM) | 1.0 µL |
| rGST-F1 (10 µM) | 1.0 µL |
| Platinum PCR Supermix High Fidelity（Invitrogen） | 22.5 µL |

| **Table S8 First-round reaction condition of 3′RACE PCR** | |
| --- | --- |
| Initial denaturation | 94°C 2 min |
| 5 cycles | 94°C 30 sec, 72°C 30 sec |
| 5 cycles | 94°C 30 sec, 70°C 30 sec |
| 25 cycles | 94°C 30 sec, 66°C 30 sec |

| **Table S9 Second-round reaction system of 3′RACE PCR** | |
| --- | --- |
| Reagent | Volume (25 µL) |
| First round product of 3′RACE PCR | 0.5 µL |
| 3 GeneRacer Inner primer (10 µM) | 1.0 µL |
| rGST-F2 (10 µM) | 1.0 µL |
| Platinum PCR Supermix High Fidelity | 22.5 µL |

| **Table S10 Second-round reaction condition of 3′RACE PCR** | |
| --- | --- |
| Initial denaturation | 94°C 2 min |
| 30 cycles | 94°C 30 sec, 66°C 30 sec |
