# Supplementary material for: Cloning, phylogenetic research, and prokaryotic expression study of the metabolic detoxification gene EoGSTs1 in Empoasca onukii Matsuda
Source: PeerJ. 2019 Sep 6;7:e7641. doi: 10.7717/peerj.7641 (PMC6733243; doi:10.7717/peerj.7641)
Supplement: Dataset S1 [file peerj-07-7641-s002.docx]

**The full cDNA fragment of EoGSTs1 gene**

**>EoGSTs1**

GGAATACACAGCATATAGCTAAATCAGTTAGATTACACTGACTATACTTGAGCTTGCAGTGTTGGTTAGTCTTTCATGTACAGTGCATCTAACAGTAACATGGCAGCTAAGTATAAAGTCACTTATTTCCCTATCATGGGGCTTGGAGAACCCATAAGATTCCTGTTGGCTTACATGGGAGAGGATTTTGAAGACTGCAGAGTCAAATGGGGTGAATGGCCTGACATAAAACCAAATACGCCGTGGGGCAAAATGCCGATTCTTGAGATCGAGGGTAAGGCAGGAGTGACCCAGAGTCAAGCAGTGACCAGGTTCTTGGCCAGACAAGCAGGGCTGTGTGGGGACGGAGCTTGGGAGGATCTCAAGATTGACGAGATCGTGTCAGTCGTTGATGAATTGAGAGGGGAGTTGGCGAAATACTACTACGAGCGTGATGAAGAGAGGAAAGCAAGTTTGAAAGAACCAGTTCTCACTCAAACTGTTCCATTTTACATGAAGAAAATAAACACTTTGATTCAAGAAAACAAAGGCTACTTGGCTAATGGAAAGTTCTCCTGGGCAGATGTGTTCTTTGCAGCGATCAGTGATCACATGAGCAACATGAACGGGTCAGATATCACGGCTGACTATCCTCAAGCCAAAGCCCTGCGAGAAAGGGTCTATGCCATCCCCAAGATAAAGGCTTGGGTGGACAAACGACCCAAAGATGTCCCCATGTTTTGAATGCCAAAATAAGACATCTTAAACCTGAGATGTTAACTGAAATTGAAATATTTATCTCACAAAACTAATTGTTCTGCATTAATAAATAAGGTTTAATTCACTAAAAAAAAAAAAAAAA

**The deduced amino acid sequence:**

> **EoGSTS1**

MAAKYKVTYFPIMGLGEPIRFLLAYMGEDFEDCRVKWGEWPDIKPNTPWGKMPILEIEGKAGVTQSQAVTRFLARQAGLCGDGAWEDLKIDEIVSVVDELRGELAKYYYERDEERKASLKEPVLTQTVPFYMKKINTLIQENKGYLANGKFSWADVFFAAISDHMSNMNGSDITADYPQAKALRERVYAIPKIKAWVDKRPKDVPMF
