# Supplementary figures and images for: Cloning, phylogenetic research, and prokaryotic expression study of the metabolic detoxification gene EoGSTs1 in Empoasca onukii Matsuda
Source: PeerJ. 2019 Sep 6;7:e7641. doi: 10.7717/peerj.7641 (PMC6733243; doi:10.7717/peerj.7641)

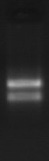

Supplement: Figure S1 [file peerj-07-7641-s006.jpg]
